# Plasma levels of soluble TGF β receptor type III: no apparent promise as a marker in acute pancreatitis

**DOI:** 10.3325/cmj.2021.62.264

**Published:** 2021-06

**Authors:** Grgur Salai, Marko Zelenika, Stela Hrkač, Vladimir Trkulja, Joško Bilandžić, Ivica Grgurević, Ruđer Novak, Lovorka Grgurević

**Affiliations:** 1 University of Zagreb School of Medicine, Zagreb, Croatia; 2 Department of Gastroenterology, Dubrava University Hospital, Zagreb, Croatia; 3 Division for Liver Diseases, Department of Gastroenterology, Dubrava University Hospital, Zagreb, Croatia; 4 Laboratory of Mineralized Tissues, University of Zagreb School of Medicine, Zagreb, Croatia

## Abstract

**Aim:**

To assess the potential of the soluble transforming growth factor β receptor type III (sTGFβrIII), a key regulator in TGFβ signaling, as a biomarker for diagnosis and stratification of patients with acute pancreatitis (AP).

**Methods:**

In this small prospective pilot study, patients’ (N = 22) plasma samples were obtained at three time points: the first and fourth day of hospitalization and the day of hospital discharge. Healthy controls’ plasma (N = 25) was obtained at a single time point. Concentration of sTGFβrIII in plasma was determined by ELISA. Data were analyzed by fitting linear or linear mixed models.

**Results:**

Plasma sTGFβrIII levels at presentation (day 1) were similar in AP patients and healthy participants, irrespectively of the disease severity. sTGFβrIII levels in patients were constant during hospital stay.

**Conclusion:**

These observations do not support further evaluation of plasma sTGFβrIII levels in this setting, but do not exclude a potential biological role of TGFβ and membrane-bound TGFβrIII in AP pathophysiology.

Acute pancreatitis (AP) is an inflammatory condition of the pancreas most commonly caused by bile stones or excessive alcohol use (1). It has a wide spectrum of presentations – from mild (most commonly) to life threatening – and may trigger a systemic inflammatory response that could lead to organ dysfunction. An accurate and timely diagnosis and risk stratification are critical for treatment and the optimization of follow-up. This might be of a particular interest in initially milder-to-moderate forms of the disease that could deteriorate over subsequent days (2). Risk stratification in AP is an ongoing challenge considering the limitations of current prognostic scores, which are predominantly based on clinical and radiological findings. Although certain biochemical indicators are essential for diagnosis (serum amylase and lipase), they are without or of limited predictive value (C-reactive protein [CRP], procalcitonin) (2,3). Some cytokines found in plasma, such as interleukin 6 or 8, show promise in severity discrimination, however, they are not routinely used in clinical practice for this indication (4,5).

Transforming growth factor β (TGF β) is a pleiotropic cytokine involved in the regulation of vital cellular processes (eg, maturation and differentiation; cell homeostasis and/or death) (6) as well as in the pathophysiology of malignant diseases, inflammation, and autoimmunity (7-9). TGFβ mediates its signaling mainly through TGFβ receptor type III (TGFβrIII), a homodimeric co-receptor that facilitates signal transduction by promoting ligands to the type II TGFβ receptor without intrinsic kinase activity (6,8). Unlike other TGFβ receptors, it is abundantly expressed on almost every human cell type (8,10). TGFβrIII generates, possibly via ectodomain shedding, a soluble form of the receptor (sTGFβrIII) (11-13), a potent TGFβ neutralizing agent with a confirmed presence in plasma (14-17). The connection between TGF-β and inflammation is a complex one (6,18). It seemingly involves TGFβrIII, and might be context-dependent, similarly to the role of TGF-β in cancer formation and progression (16,19-22). Generally, TGFβ is a strong anti-inflammatory cytokine. Disruption of its signaling results in an increased T-cell response (23), and TGFβrIII has been implicated in Th 17 lymphocyte (CD4+ and CD8+) activation (20,24). In relation to AP specifically, TGFβrIII mRNA was found to be moderately increased in AP tissue samples (25). Taken together, it appears plausible to assume that the plasma levels of the soluble form – sTGFβrIII – might be a biochemical marker in AP. To investigate the feasibility of this hypothesis, we conducted a pilot study in patients with mild-to-moderate AP.

## Patients and methods

### Study outline

This prospective observational study enrolled consecutive adults diagnosed with a first episode of AP graded as “mild” or “moderate,” admitted between December 10, 2019 and February 18, 2020 at a single tertiary center (University Hospital Dubrava), and a sample of generally healthy volunteers. Patients provided blood samples for sTGFβrIII determination at presentation to the hospital (day 1), day 4 of hospital stay, and at discharge, while healthy participants provided a single blood sample for this purpose. The third time point (discharge day) was purposely chosen not to be a “fixed” day of hospital stay, but rather to represent significant clinical improvement – patients were discharged when the following was achieved: improvement of symptoms or no symptoms reported; adequate oral feeding; and no systemic complications and partial improvement or resolution of local complications. All participants provided a signed informed consent. The study was approved by the Ethics Committee of Dubrava University Hospital.

### Participants

All participants had to be free of any other acute or chronic inflammatory disease and have no medical history of malignancy. Acute pancreatitis was diagnosed and classified in line with the revised Atlanta criteria (26), and treatment was in line with the International Association of Pancreatology and American College of Gastroenterology guidelines for the management of AP (27).

### sTGFβrIII measurement

Peripheral blood was drawn into citrated Vacutainer tubes (citrate to blood 1:9); plasma was immediately separated by centrifugation (15 minutes at 3000 g) and was kept at -80 °C until analysis. An indirect ELISA kit (Human TGF-beta RIII DuoSet DY242, R&D, Minneapolis, MN, USA) was used to determine the plasma sTGFβrIII expression levels according to the manufacturer's instructions. All samples and standards were analyzed in duplicates, and the samples with an individual coefficient of variation (CV) greater than 25% were retested in duplicates.

### Data analysis

Data are summarized by health status, AP severity, and time point, and were analyzed by fitting linear or linear mixed models (SAS for Windows 9.4, SAS Inc., Cary, NC, USA). Where required, the variables were transformed to achieve normality of residuals.

## Results

**Figure 1:**
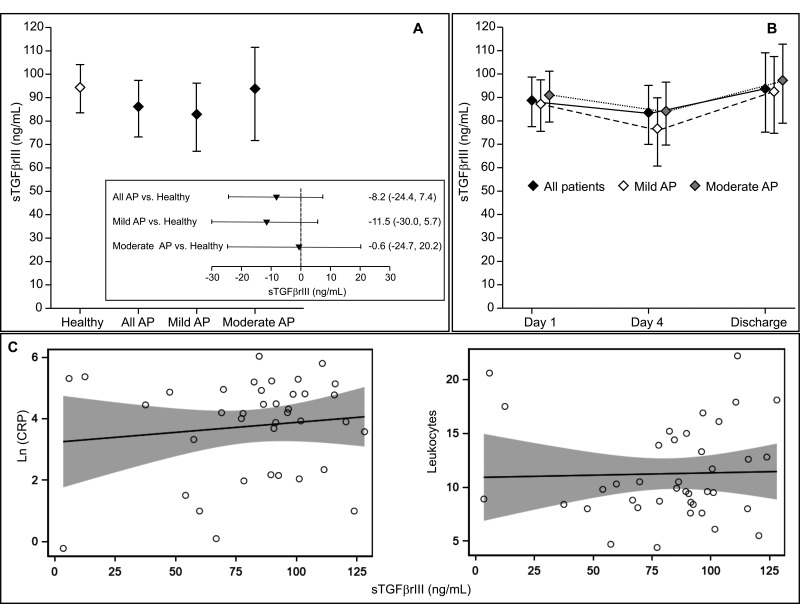
(**A**) Age and sex-adjusted mean (95% confidence interval, CI) plasma soluble type III transforming growth factor β receptor (sTGFβrIII) levels in healthy participants and patients with acute pancreatitis (AP) (overall and by severity classified in line with the revised Atlanta criteria). The insert depicts adjusted differences between participants, with 95% CIs. A general linear model (effects: age, sex, health condition) was fitted to sTGFβrIII levels to generate adjusted means and mean differences. (**B**) Adjusted sTGFβrIII levels (mean, 95% CI) in AP patients over the observed period (overall and by severity). A mixed model (fixed effects: age, sex, time, disease severity and time*severity interaction) was fitted to sTGFβrIII. (**C**) Relationship between plasma sTGFβrIII levels and C-reactive protein (left) or leukocyte counts (right). A separate mixed model (fixed effects: age, sex, time, sTGFβrIII, time* sTGFβrIII interaction) was fitted to (ln) C-reactive protein and leukocyte count to generate depicted adjusted regression lines (shaded area: 95% CIs). The same analysis was conducted with serum lipases and neutrophil-to-lymphocyte ratio (not shown), also showing no association with sTGFβrIII.

A total of 22 AP patients (predominantly biliary AP; 15 mild, 7 moderate AP) and 25 healthy participants were included (Table 1). Plasma sTGFβrIII levels at presentation (day 1) were similar in AP patients and healthy participants, irrespectively of the disease severity (Table 1, Figure 1A). C-reactive protein levels, serum amylase, lipase, leukocyte counts, and neutrophil-to-lymphocyte ratio gradually decreased until hospital discharge (Table 1), while sTGFβrIII levels appeared constant over time (Table 1, Figure 1B). There was no association between plasma sTGFβrIII levels and CRP, leukocyte counts, or neutrophil-to-lymphocyte ratio (not shown). Individual patients’ characteristics are available in the Supplementary Table 1.(Supplementary Table 1)

**Table 1:** Participants' characteristics at presentation (day 1 or the day of blood sampling for healthy participants), on day 4, and on the day of discharge. Patient data are shown overall and by Atlanta classification of acute pancreatitis (AP) severity. Data are median (Q1-Q3, also range for age and sTGFβIII) or count (percent)*

	All patients	Mild AP	Moderate AP	Healthy participants
N	22	15	7	25
Men	13 (59.1)	8 (53.3)	5 (71.4)	15 (60.0)
Age (years)	62 (54-68; 31-79)	61 (44-63; 31-79)	63 (54-75; 53-78)	48 (31-61; 20-80)
Idiopathic AP	2 (9.1)	2 (13.3)	0	—
Alcohol-related AP	4 (18.2)	3 (20.0)	1 (14.3)	—
Biliary AP	15 (68.2)	9 (60.0)	6 (85.7)	—
Hypertriglyceridemia	1 (4.5)	1 (6.7)	0	—
**Day 1**				
sTGFβrIII (ng/mL)	89.5 (65.0-103; 5.9-128)	85.5 (59.9-98.5; 5.9-124)	96.6 (778-116; 12.5-128)	91.6 (71.9-110; 20.2-128)
Modified Glasgow score	1 (1-1.75)	1 (1-1.25)	1 (1-2)	—
BISAP score				—
0	7 (31.8)	6 (40)	1 (14.3)	—
1	8 (36.4)	7 (46.67)	1 (14.3)	—
2	5 (22.7)	1 (6.67)	4 (57.1)	—
3	2 (9.1)	1 (6.67)	1 (14.3)	—
APACHE II score	5 (2.5-7.5)	5 (2-7)	5 (5 - 8)	—
Amylase (IU/L)	595 (357-1687)	753 (514-1700)	411 (276-511)	—
Lipase (IU/L)	1944 (715-2943)	1939 (640-2626)	2336 (1186-3110)	—
CRP (mg/L)	70.4 (8.5-145)	66.3 (4.5-137)	74.5 (50.3-129)	—
Leukocytes (x 10^9^/L)	12.6 (9.6-16.9)	10 (9.6-14.6)	13.9 (11.1-17.2)	—
NLR	8.7 (5.6-15.5)	8.4 (5.62-13.3)	9.5 (8.58-21.4)	—
**Day 4**				
sTGFβrIII (ng/mL)	86,3 (69.7-96,2)	77.7 (50-87.5)	101 (91-103)	—
Amylase (IU/L)	89.5 (79.5-165)	92 (79-172)	87 (85-98)	—
Lipase (IU/L)	134 (69-192)	103 (57-190)	198 (166-229)	—
CRP (mg/L)	87 (44-135)	60.6 (22.9-132)	118 (76.1-151)	—
Leukocytes (x 10^9^/L)	8.7 (7.6-11.1)	10.1 (9.6-14.6)	13.9 (11.1-17.2)	—
NLR	5.4 (2.7-6.4)	5.85 (3.47-9.53)	2.72 (1.91-3.29)	—
**Discharge (5-13 days post admission, 1 patient day 35)**				
sTGFβrIII (ng/mL)	85,1 (76.7-95.6)	83.3 (70.9-86.6)	99.5 (89.8-104)	—
Amylase (IU/L)	81.5 (73.5-87)	82 (81-87)	75 (72-81)	—
Lipase (IU/L)	66 (53-67)	66 (59.5-66.5)	91 (69.5-113)	—
CRP (mg/L)	20.6 (8.15-41)	22.1 (6.4-49)	22 (16.6-25.5)	—
Leukocytes (x 10^9^/L)	6.6 (5.83-9.35)	6.15 (5.15-7.93)	8.35 (6.4-11.3)	—
NLR	2.92 (2.12-4.54)	3.11 (2.46-4.46)	2.73 (2-7)	—

*Abbreviations: AP – acute pancreatitis; APACHE II – Acute Physiology And Chronic Health Evaluation II; BISAP – bedside index of severity in acute pancreatitis; CRP – C-reactive protein; NLR – neutrophil-to-leukocyte ration; sTGFβrIII – soluble type III transforming growth factor β receptor.

## Discussion

The need for (additional) readily available biochemical markers that would be useful aids to guide treatment and follow-up procedures in AP patients has been well recognized (2,3), and a number of candidates have been suggested to date (3). Biologically, sTGFβrIII appears to be a plausible potential candidate: TGFβ and TGFβrIII are known to be involved in inflammatory events, TGFβrIII has been implied specifically in AP (based on tissue expression), and ectodomain shedding (source of plasma sTGFβrIII) as a part of acute response to noxious stimuli has been documented using transmembrane TGFα as a model (6,20,24,28). The underlying concept implies that noxious stimuli induce the activity of ectodomain sheddases resulting in a release of sTGFβrIII – a process that is upregulated by proinflammatory cytokines (29). The characterization of a reliable biochemical indicator in any setting is a complex time- and resource-consuming task (30); we therefore considered it reasonable to conduct a preliminary evaluation of plasma sTGFβrIII levels “response” to milder forms of AP (where a “signal” would indicate the feasibility of a more extensive evaluation). We observed no difference in sTGFβrIII levels between the affected patients at presentation and their healthy peers, no obvious dynamics in sTGFβrIII level over the course of the disease, and no relationship between sTGFβrIII and other routinely used indicators of tissue damage (enzymes) or inflammation (no indication of concurrent validity). These observations do not support further evaluation of plasma sTGFβrIII levels in this setting, but do not exclude a potential biological role of TGFβ and membrane-bound TGFβrIII in AP pathophysiology. The present observations might be a result of a number of possible underlying mechanisms, such as eg, formation of TGFβ-sTGFβrIII complexes that may not be detected by ELISA assays or the escape of the membrane-bound TGFβrIII from ectodomain shedding due to involvement in the tissue repair processes (31). It is also possible that the relationship of TGFβrIII with inflammation is more closely related to the chronic inflammatory processes as observed in cancer at the systemic level (16,32).

Overall, although limited by a small single-center sample, the present study does not support further evaluation of plasma sTGFβrIII levels as a potential diagnostic/prognostic aid in acute pancreatitis.
