# Supplementary material for: Plasma levels of soluble TGF β receptor type III: no apparent promise as a marker in acute pancreatitis
Source: Croat Med J. 2021 Jun;62(3):264–9. doi: 10.3325/cmj.2021.62.264 (PMC8275940; doi:10.3325/cmj.2021.62.264)
Supplement: Supplementary Table 1 [file CroatMedJ_62_s001.pdf]

Supplementary Table S1. Inidividual patients' clinical characteristics.

General information			AP type	Comorbidities								Diagnosis			Details during hospitalization						Clinical assessment			sTGFβrIII			Length of stay
Patient no.	Sex	Age		Liver disease	DM	CVD	Dyslipidemia	Hypertension	CKD	Smoker	Alcoholic	Clinical	Enzymes	Imaging	Imaging during hosp.	Antibiotics	LMWH	Most likely etiology	Local complications	Systemic complications	Day 1	Day 4	Discharge day	Day 1	Day 4	Discharge day	
1	♂	54	mild	no	no	no	no	yes	no	yes	yes	yes	yes	yes	MSCT	no	no	alcohol	no	no	mdrt	mild	No*	68.97	84.53	73.93	12
2	♂	62	mild	no	yes	yes	yes	yes	yes	no	no	yes	yes	no	US, EUS, MSCT	yes	no	biliary	no	no	mild	No*	No*	110.66	86.3	92.68	7
3	♀	36	mild	no	no	no	no	no	no	yes	no	yes	yes	yes	US, EUS	no	no	biliary	no	no	mild	No*	No*	59.86	3.49	86.59	8
4	♂	31	mild	no	no	no	no	no	no	no	yes	yes	yes	no	US	no	no	alcohol	no	no	mild	No*		123.83	120.44		*
5	♂	70	mild	no	no	yes	no	yes	no	no	no	yes	yes	yes	US, EUS	yes	yes	biliary	no	no	mdrt	No*	No*	98.55	77.16	105.76	12
6	♀	79	mild	no	no	yes	yes	yes	no	no	no	yes	yes	yes	US	yes	yes	biliary	no	no	mdrt	mdrt	No*	85.45	96.24	70.86	9
7	♀	53	mdrt	no	no	no	no	no	no	no	no	yes	yes	yes	US, EUS, MSCT	yes	yes	biliary	APFC	Pl.ef, resp. in.	svr	svr	No*	12.45	100.63	96.59	35
8	♂	63	mild	no	no	no	no	yes	no	no	no	yes	yes	no	US, EUS	yes	yes	biliary	no	no	mdrt	No*	No*	89.69	69.74	83.32	7
9	♀	41	mild	no	no	no	no	no	no	no	no	yes	yes	yes	US, EUS, CT	yes	no	biliary	no	no	mdrt	No*	No*	66.75	78.2	39.71	13
10	♀	61	mild	no	yes	no	yes	yes	no	no	no	yes	yes	yes	MSCT	yes	yes	unk	no	no	mdrt	No*	No*	37.51	57.46	68.38	10
11	♀	54	mild	no	no	no	no	no	no	yes	no	yes	yes	yes	US, EUS	no	no	biliary	no	no	mdrt	No*	No*	5.89	0	0	6
12	♀	62	mild	no	no	no	yes	yes	no	no	no	yes	yes	yes	UZV, EUS, CT	yes	yes	biliary	no	no	mdrt	mdrt	mild	74.45	33.06	83.4	9
13	♂	75	mdrt	no	no	yes	no	yes	no	no	yes	yes	yes	yes	MSCT, EUS	yes	yes	biliary	APFC	no	mdrt	mdrt	mild	96.65	82.42	87.585	11

General information			AP type	Comorbidities								Diagnosis			Details during hospitalization						Clinical assessment			sTGFβrIII			Length of stay
Patient no.	Sex	Age		Liver disease	DM	CVD	Dyslipidemia	Hypertension	CKD	Smoker	Alcoholic	Clinical	Enzymes	Imaging	Imaging during hosp.	Antibiotics	LMWH	Most likely etiology	Local complications	Systemic complications	Day 1	Day 4	Discharge day	Day 1	Day 4	Discharge day	
14	♂	58	mild	no	no	no	yes	yes	no	yes	yes	no	yes	yes	US, MSCT	no	yes	trigl.	no	no	mild	No*	No*	89.38	87.95	79.54	13
15	♂	44	mild	no	no	no	no	no	no	yes	no	yes	yes	no	US, MSCT	yes	yes	unk	no	no	mdrt	No*	No*	96.07	92.53	94.66	5
16	♀	79	mild	no	no	yes	no	yes	no	no	no	yes	yes	yes	US, EUS, MSCT	yes	no	biliary	no	no	mild			111.36			*
17	♀	62	mdrt	no	yes	yes	yes	yes	no	no	no	yes	yes	no	MSCT, US,	yes	yes	biliary	APFC	no	mdrt	No*	mild	128.1	103.46	103.9	8
18	♂	62	mild	no	no	no	no	yes	no	no	yes	yes	yes	no	US, MSCT	no	no	biliary	no	no	mdrt	mild	No*	54.07	47.52	85.06	8
19	♂	63	mdrt	no	no	no	no	yes	no	no	no	yes	yes	no	MSCT, US	yes	yes	biliary	APFC	no	svr	mdrt	mild	77.85	91.36	84.61	9
20	♂	54	mdrt	no	no	no	no	no	no	yes	yes	yes	yes	no	MSCT, US, EUS	no	no	alcohol	APFC	no	mdrt	mild	No*	91.53	90.62	102.32	9
21	♂	78	mdrt	no	no	no	no	yes	no	no	yes	yes	no	yes	MSCT, US	no	no	alcohol	APFC	no	mild	mild	No*	115.97	115.63	113.48	8
22	♂	67	mdrt	no	no	yes	no	yes	no	no	no	yes	yes	yes	MSCT, US, EUS	yes	yes	biliary	APFC	no	mdrt	mild		101.105	101.75		*

Supplementary table includes: General information including patient number, sex (♂ - male, ♀ - female); age in years; type of acute pancreatitis according to the Revised Atlanta severity (*AP type*: mild, *mdrt* – moderate). Comorbidities including: liver disease, diabetes mellitus (DM), cardiovascular diseases (*CVD*), dyslipidemia, hypertension, chronic kidney disease (*CKD*), smoking status (*smoking*) and whether the patient is known to suffer from alcohol abuse (*alcohol*). Data on how the diagnosis was made, according to the Revised Atlanta Criteria: typical clinical presentation (*Clinical*), increase of serum lipase or amylase levels >3x the upper limit (*Enzymes*), typical findings on radiological imaging (*Imaging*). This table also present details during hospitalization which include the imaging used during hospital stay (*Imaging during hosp.*): *US* – abdominal ultrasound, *EUS* – endoscopic ultrasound, *MSCT* – multislice computer tomography, data on whether antibiotics (*Antibiotics*) or low molecular weight heparin (*LMWH*) were prescribed; as well as most likely etiology of acute pancreatitis: *alcohol* – alcohol-related; *unk* – unkown; *trigl.* – hypertriglyceridemia. This section also contains local and systemic complications observed during hospital stay (*APFC* – acute peripancreatic fluid collection; *pl. ef.*- pleural effusion; *resp. in.* – respiratory

insufficiency). Clinical assessment performed by a board certified gastroenterologist was stratified into 4 subcategories: *No** - patient has no symptoms; *mild* – patient has bearable symptoms and/or mild clinical presentation; *mdrt* – moderate: patient has „typical" clinical presentation, is in pain and requires analgesia; *svr* – severe: patient has severe clinical presentation and/or is in severe pain not managable with standard therapy. sTGFβrIII concentration in plasma is in ng/mL. The last column (*Length of stay*) represents duration of hospital stay in days; * - patient declined participation in the final time point of the study and/or left hospital against medical advice. Blank fields signify absence during the appropriate time point. **Patient managment summary:** Patients who matched the inclusion criteria were initially evaluated in the Emergency Department. They were assessed for signs of severe acute pancreatitis and organ failure or signs of systemic inflammatory response syndrome. Clinically relevant scores such as APACHE II and BISAP were measured. In case of unclear diagnosis a further imaging study was performed. Depending on the severity of the pancreatitis they were admitted to the intensive care unit or general hospital ward where they were treated by a board certified gastroenterology specialist. Treatment was guided by relevant society guidelines such as International Association of Pancreatology (IAP) and American College of Gastroenterology (ACG) guidelines for management of acute pancreatitis. Early goal-directed fluid resuscitation was given with frequent fluid requirement assessment. Pain control was achieved with NSAIDs or opioid analgesics (intravenous or oral tramadol and morphine). Antibiotics were only used in cases or signs of extrapancreatic infections such as cholecystitis, cholangitis, pneumonia or urinary tract infection. Early oral refeeding was started as soon as it was tolerated and in more severe cases a nasogastric delivery was used. Patients with cholangitis and ongoing biliary obstruction had an early ERCP (within 24 hours of admission) procedure with removal of the gallstones. In cases where there were no signs of cholangitis but a high risk of gallstone pancreatitis was present, patients were further evaluated with magnetic resonance cholangiopancreatography (MRCP) or endoscopic ultrasound (EUS) and ERCP was done if needed. During the hospital stay all patients were monitored with regular clinical evaluation and laboratory studies. A follow-up US or MSCT was done in case of moderately severe pancreatitis to assess the development of local complications. Patients were discharged when the following was achieved: improvement of symptoms or no symptoms reported; adequate oral feeding; no systemic complications and partial improvement or resolution of local complications. Patients with proven gallstone pancreatitis were referred to an abdominal surgeon for early cholecystectomy.
